# Remodeling of Rat M. Gastrocnemius Medialis During Recovery From Aponeurotomy

**DOI:** 10.3389/fphys.2020.541302

**Published:** 2020-10-28

**Authors:** Cintia Rivares, Reinald Brunner, Johan J. M. Pel, Guus C. Baan, Peter A. Huijing, Richard T. Jaspers

**Affiliations:** ^1^Laboratory for Myology, Bewegingswetenschappen, Vrije Universiteit Amsterdam, Amsterdam, Netherlands; ^2^Pediatric Orthopaedic Department, Children’s Hospital, University of Basel, Basel, Switzerland; ^3^Department of Neuroscience, Erasmus Medisch Centrum (MC), Rotterdam, Netherlands

**Keywords:** adaptation, aponeurosis, force transmission, intramuscular aponeurotic recession, muscle release, muscle lengthening, sarcomeres in series

## Abstract

Aponeurotomy is a surgical intervention by which the aponeurosis is transsected perpendicularly to its longitudinal direction, halfway along its length. This surgical principle of aponeurotomy has been applied also to intramuscular lengthening and fibrotomia. In clinics, this intervention is performed in patients with cerebral palsy in order to lengthen or weaken spastic and/or short muscles. If the aponeurotomy is performed on the proximal aponeurosis, as is the case in the present study, muscle fibers located distally from the aponeurosis gap that develops lose their myotendinous connection to the origin. During recovery from this intervention, new connective (scar) tissue repairs the gap in the aponeurosis, as well as within the muscle belly. As a consequence, the aponeurosis is longer during and after recovery. In addition, the new connective tissue is more compliant than regular aponeurosis material. The aim of this study was to investigate changes in muscle geometry and adaptation of the number of sarcomeres in series after recovery from aponeurotomy of the proximal gastrocnemius medialis (GM) aponeurosis, as well as to relate these results to possible changes in the muscle length-force characteristics. Aponeurotomy was performed on the proximal aponeurosis of rat muscle GM and followed by 6 weeks of recovery. Results were compared to muscles of a control group and those of a sham-operated group. After recovery from aponeurotomy, proximal and distal muscle fiber lengths were similar to that of the control group. The mean sarcomere length from fibers located proximally relative to the aponeurosis gap remained unchanged. In contrast, fibers located distally showed 16–20% lower mean sarcomere lengths at different muscle lengths. The number of sarcomeres in series within the proximal as well as distal muscle fibers was unchanged. After recovery, muscle length-force characteristics were similar to those of the control group. A reversal of proximal-distal difference of fibers mean sarcomere lengths within muscles during recovery from aponeurotomy is hypothesized to be responsible for the lack of an effect. These results indicate that after recovery from aponeurotomy, geometrical adaptations preserved the muscle function. Moreover, it seems that the generally accepted rules of adaptation of serial sarcomere numbers are not applicable in this situation.

## Introduction

Aponeurotomy is a surgical intervention aimed at restoring the range of joint motion. This surgical principle is applied in technical modifications such as intramuscular lengthening and fibrotomia. This intervention is executed for the triceps surae muscle by one or more transections of the aponeurosis perpendicular to its length (Majestro et al., 1971; Baumann and Koch, 1989) and is performed in patients with spastic cerebral paresis (Baddar et al., 2002). The clinical aim is either lowering of muscle force and/or lengthening of the muscle(s), which are likely involved in limiting the range of joint motion (Nather et al., 1984; Baddar et al., 2002). Although this intervention has been shown to be successful initially, in approximately 15–48% of the patients recurrence of the restricted range of foot dorsal flexion was reported (Olney et al., 1988; Saraph et al., 2000).

Experimental aponeurotomy, performed on rat gastrocnemius medialis (GM) and extensor digitorum longus muscles, has shown that excitation of the muscle and stretch acutely after aponeurotomy results in a spontaneous rupture of the intramuscular connective tissue below the location of aponeurotomy (Jaspers et al., 1999, 2002). This feature is also known clinically in children with equines feet, at least by those surgeons performing aponeurotomies. As a consequence, lengths of muscle fascicles and fibers, being continuous with the origin or insertion at only one end, are substantially reduced (Jaspers et al., 1999, 2002). Moreover, rupturing of the intramuscular connective tissue is accompanied by an acute reduction in muscle force of approximately 50%, as well as an increase of optimum muscle length (by 10%; Jaspers et al., 1999, 2002). Such effects of aponeurotomy are favorable in (partially) restoring acutely a previously restricted range of joint motion.

Histological analysis indicated that during recovery, new connective tissue is laid down reconnecting the two aponeurotic ends and filling the gap below (Brunner et al., 2000; Jaspers et al., 2005). As a consequence, also after recovery from aponeurotomy, the proximal aponeurosis of GM is longer and also more compliant than in untreated muscle (Jaspers et al., 2005). This implies that muscle fibers attaching to the proximal aponeurosis, but distally from the new tissue, will be kept much shorter than before aponeurotomy, with possible consequences for the length-force characteristics (due to enhanced distribution of fiber mean sarcomere length within the muscle; e.g., Willems and Huijing, 1994) and number of sarcomeres in series within muscle fibers.

For parallel fibered muscle, it is generally acknowledged that *in vivo* the number of sarcomeres arranged in series within a muscle fiber adapts to the length at which a muscle is mostly active (Williams and Goldspink, 1978; Herring et al., 1984; Heslinga and Huijing, 1993; Heslinga et al., 1995). For parallel fibered muscle, adaptation of optimum muscle length is achieved primarily by changes in the serial number of sarcomeres. However, it should be noted that for highly pennate muscles (as GM), aspects of muscle fiber diameter may contribute to altered optimum length, possibly taking away or diminishing the signal for serial sarcomere adaptation (cf. Swatland, 1980; Heslinga and Huijing, 1993; Huijing and Jaspers, 2005). Post aponeurotomy, it is conceivable that distal muscle fibers will have a decreased number of sarcomeres in series with implications for the length-force characteristics. The question is raised whether pennate GM, after recovery from aponeurotomy, shows such adaptation and/or geometric remodeling and how these features relate to the possible muscle length-force properties. Therefore, the aim of the present study was to investigate muscle geometry and fiber mean sarcomere lengths and its distribution after a 6-week period of recovery from aponeurotomy in rat muscle (m.) GM and to relate these to possible changes in muscle length-force characteristics.

## Materials and Methods

### Animals and Experimental Protocol

**Figure 1:**
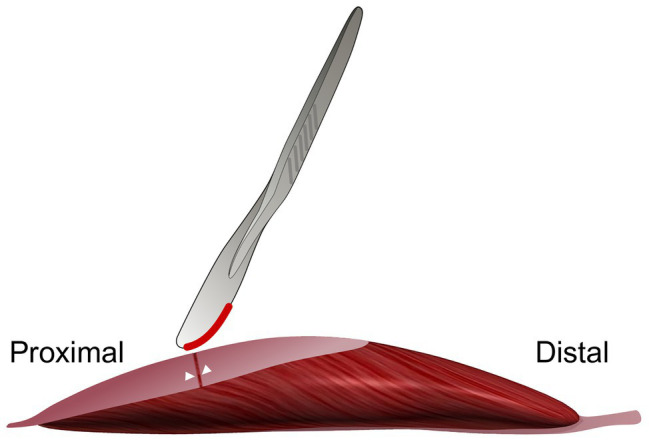
Schematic figure illustrating the aponeurotomy intervention of the proximal gastrocnemius medialis (GM) aponeurosis. The proximal aponeurosis of the GM in the right limb was cut at its middle perpendicular to its length. The white markers indicate the locations where the 0000 silk sutures were placed. Note that the GM muscle is illustrated free from other muscles and structures. *In vivo*, during the intervention, surrounding muscles are present.

**Figure 2:**
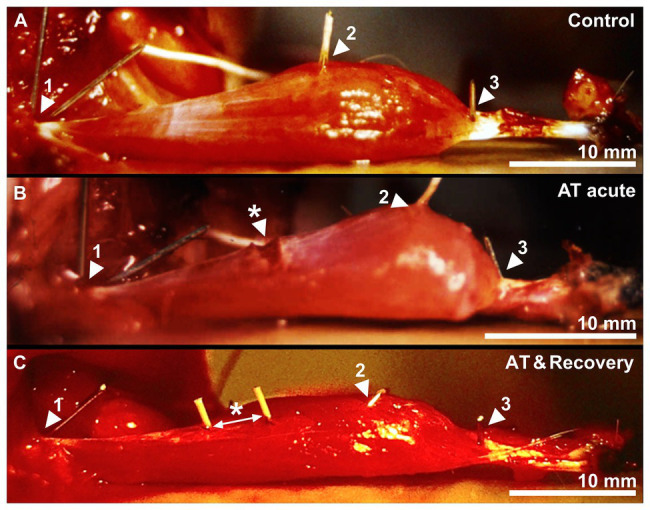
A control, acutely aponeurotomized and a recovered muscle after aponeurotomy. **(A)** Active muscle near optimum length of a control GM muscle. **(B)** The effects of acute aponeurotomy on the muscle. The fibers of the aponeurosis were transsected, creating a gap on the medial aponeurosis. **(C)** Active muscle near optimum length of a recovered GM muscle. Markers 1, 2, and 3 represent the origin of the muscle, the distal end of the proximal aponeurosis, and the distal end of the distal muscle fiber, respectively. The white arrow indicates the space between the markers, which were placed at the location of the sutures. * indicates the newly formed connective tissue, closing the aponeurotic gap.

All surgical and experimental procedures were performed in strict agreement with Swiss law and regulations concerning animal welfare. Experiments were performed on the right m. GM of male Wistar rats. Three experimental treatment groups of animals are distinguished:

1. Aponeurotomy was performed on the proximal aponeurosis of GM of young adult Wistar rats [*n* = 6, mean age 9.1 weeks, body mass 373.3 ± 7.2 g (mean ± SEM)]. The proximal aponeurosis of the right limb was transsected at its middle perpendicular to its length direction (Figure 1) using a scalpel blade (number 23). Moreover, the proximal aponeuroses of all remaining parts of the right m. triceps surae were also transsected during the intervention. The location of the transection of the aponeurosis was marked by silk 0000 sutures at each end of the aponeurotic parts at the side of the transection (Figure 2).
  After the intervention, a walking cast was applied to the right hind limb to maintain maximal dorsiflexion. The walking cast was removed 4 days after the intervention. After 6 weeks of recovery, length-force data were collected for the maximally dissected *in situ* GM. For this, the muscle was freed of surrounding myofascial connective tissues, leaving only blood vessels, lymphatics, and its connection to only the distal end of its nerve with their connective tissue reinforcements intact. Particularly in the figures, this group of animals is referred to as “AT &recovery.”
2. A sham operation was performed on the right limb of young adult rats [*n* = 6, mean age 9.1 weeks, body mass 383.8 ± 8.4 g (mean ± SEM)]. This intervention implies opening of the skin and performing blunt dissection of the connective tissue surrounding the muscle, similar as done for the AT recovery group. The length of the aponeurosis of the GM was measured with the ankle flexed at a right angle and its center marked with a silk 0000 sutures. Subsequently, a lower-limb walking cast was applied to the right limb to maintain maximal dorsiflexion. After 6 weeks of recovery, length-force characteristics were determined of the maximally dissected *in situ* GM. This group of animals is referred to as “sham.”
3. Control group. The right limb of animals of this group [*n* = 10, mean age 14.9 weeks, body mass 357.1 ± 3.6 g (mean ± SEM)] was not exposed to any surgery or casting. After 6 weeks, length-force data were collected of the intact but maximally dissected *in situ* GM. Operations, application, and removal of cast were performed under general anesthesia (halothane 0.5–2%, head mask). For a postoperative period of 3 days, the animals were kept under paracetamol (200 mg kg^−1^ per day) as analgesic. The animals were housed under standard conditions.

### Physiological Experiments

Animals were anesthetized by intraperitoneal injection of sodium pentobarbital (initial dose 8 mg/100 g body mass) and ventilated mechanically. The medial head of GM of the right limb was dissected, leaving its origin intact. The blood supply was left undisturbed, whereas the sciatic nerve was cut as proximally as possible. The Achilles tendon with a piece of calcaneal bone still attached was connected by a metal wire to a force transducer (A&D Company LC-4101). Small copper markers were inserted into the muscle to mark the origin of the muscle, the distal end of the proximal aponeurosis, and the distal end of the distal fiber (Figure 2).

The animal was positioned in the experimental setup by rigidly clamping the femur. Ambient temperature was controlled at 27°C. The proximal end of the nerve was stimulated supramaximally (100 Hz, 0.15-ms square wave pulse) using silver electrodes connected to a constant current source. The necessary electrical voltage was determined individually per muscle. Muscles were lengthened at a muscle length at which 1.9 V produced less than 0.05 N. Following this, the voltage was incremented by 0.05 V until maximum active force was produced (i.e., further increasing the voltage yielded lower active muscle forces). The necessary electrical voltage was between 1.75 and 5.00 V. Tetanic isometric contractions of 600 ms were induced at a series of muscle lengths, beginning near active slack length and ending at the length at which force was approximately 90% of maximum force (1-mm increments). Each tetanic contraction was preceded by two twitches with a 1-s interval to allow adjustment of the muscle to that length. Three seconds after the last twitch, a tetanic contraction was evoked. The interval between two subsequent tetanic contractions was 2 min, during which the muscle was allowed to recover at short length. The muscle was photographed (Canon A1 camera, macro lens, exposure time 1/125 s, 400 ASA slide film) in passive state (2 s before activation) and in fully active state (200 ms after evoking tetanic stimulation). A microcomputer was used to collect all force data, using an AD-converter (Validyne Engineering Corp. UPC601-G, sampling frequency of 1,000 Hz and resolution of force 0.0071 N).

### Dimensions of Anatomical Structures

Postexperimentally, morphological parameters were estimated by determination of the marker positions on projected photographic images (magnification 15×) using a digitizer tablet (Microgrid III Summagrafics Co., mean error 0.017 mm) and a software program (AutoCAD 12.0). Muscle lengths were determined for all muscles, and the most proximal and distal muscle fiber lengths were determined in the mid longitudinal plane of GM for all muscles. Direct measurement of the distances between the markers provides estimates of muscle length, length of proximal aponeurosis, and length of distal fiber. The lengths of the proximal muscle fibers were determined as follows. Based on findings of Zuurbier and Huijing (1993) and our own pilot data, the length of the distal aponeurosis was assumed to be 1.11 times the length of the proximal aponeurosis. The distal end of distal aponeurosis was estimated 0.5 mm below the distal end of distal fibers. Taking this point as the center, a circle of radius (length distal aponeurosis) was drawn. The longitudinal direction of the proximal part of distal aponeurosis was estimated in the image. The intersection between this direction and the circled length of the distal aponeurosis indicates the distal end of the most proximal fiber (for a detailed description, see Jaspers et al., 1999). The length of the most proximal muscle fiber was determined by the distance of between the origin of the muscle and the proximal end of the distal aponeurosis.

### Treatment of Data

Passive muscle force as a function of muscle lengths were least-squares fitted using an exponential function (Eq. 1)

(1) $$y=e^{\mathrm{b0}}{}^{x+\mathrm{b1}}+b_{2}$$

where *y* represents passive force, *x* represents muscle lengths, *b*_0_, *b*_1_, and *b*_2_ are constants determined during the fitting procedure. Active muscle force was calculated by subtracting passive force (Eq. 1) from total muscle force for the appropriate muscle lengths. The relationship of active force with muscle length was fitted by a polynomial (Eq. 2)

(2) $$y=b_{0}+b_{1}x+b_{2}x^{2}+b_{3}x^{3}+b_{4}x^{4}+b_{n}x^{n}$$

where *b*_0_ through *b*_n_ are constants selected in a least-squares fitting procedure. The lowest polynomial order that described the length-force relationship most adequately (tested by one-way ANOVA) was selected. Muscle optimum length was defined as the muscle length at which the maximum of the polynomial was encountered, within the experimental muscle length range. Muscle active slack length was determined by fitting muscle force using Eq. 1, but only for the range of muscle lengths at which active muscle force ranged between zero and 30% of maximal muscle force. Muscle active slack length was defined at the intercept of the fitted curve and the *x* axis. The relationships between the muscle fiber length, fiber mean sarcomere length, and active muscle length were fitted with a third-order polynomial, as described by Eq. 2. Fitted curves were used to calculate mean data ± SE.

### Number of Sarcomeres in Series

The numbers of sarcomeres in series in proximal and distal fibers were determined according to the method previously described by Jaspers et al. (1999). For at least 2 weeks, the muscles were fixed (4% formaldehyde, 15% absolute alcohol, and 1.5 g l^−1^ thymol). Fiber bundles taken out of the muscle were exposed to a 17.5% KOH solution (modification to Huijing 1985) for 4 h after which they were stored in a 50% glycerol solution for 2–4 days to soften connective tissue. Four fibers located proximally in the muscle, and four distally located fibers were teased out for their whole length. The number of sarcomeres in series was counted semiautomatically using a microscope (Zeiss, magnification 20β) connected to an image analyzer (low-pass filter of gray levels) counting number of A-bands every 120 μm along the fiber.

### Statistics

Two-way mixed ANOVAs (with repeated measurements for muscle length) were performed to test for differences between the muscles force, muscle fiber length, and fiber mean sarcomere length as a function of muscle length changes, as well as to test for differences within groups as function of muscle length changes.

Two-way mixed ANOVA was also used to test differences in serial sarcomere number between treatments and within muscle location (proximally vs. distally located fibers). A three-way mixed ANOVA (with repeated measurements for muscle length and muscle location) was performed to test for differences in fiber mean sarcomere length distribution between treatments. *Post hoc* comparisons were performed using the Bonferroni procedure to locate significant differences. Assumptions of the two‐ and three-way mixed ANOVAs were met. Level of significance was selected at *p* < 0.05.

## Results

### Muscle Fiber Length as Function of Muscle Belly Length After Recovery From Aponeurotomy

Note that after aponeurotomy is performed on GM proximal aponeurosis, the two muscle fiber populations are exposed to different mechanical conditions: The proximal fiber population located proximally with the muscle remains connected *via* the aponeurosis and tendon to both muscle origin and insertion (acute situation). In contrast, acutely after aponeurotomy, the distal fibers lose their connection to the origin of the muscle because of the severed proximal aponeuroses. During recovery, such contact is reestablished, due to inserting new aponeurotic tissue, albeit at a greater length than previous aponeurosis length, as well as at a higher compliance of the tissue inserted and of the whole aponeurosis (AT & recovery situation).

#### Effects of Aponeurotomy and Recovery on Proximal Muscle Fibers

**Figure 3:**
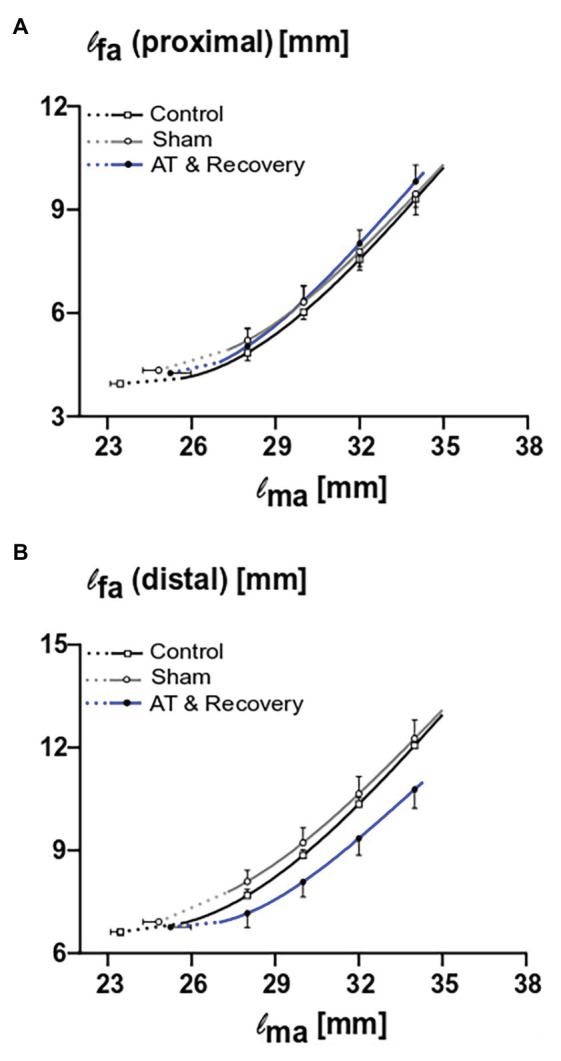
The combined effects of aponeurotomy plus recovery from aponeurotomy on length of muscle fibers. Muscle fiber lengths as function of muscle length are plotted in continuous lines, while extrapolated fiber lengths, ranging from the lowest measured muscle lengths to the estimated muscle active slack length, are plotted as dashed lines. **(A)** The length of proximal muscle fibers (l_fa_ proximal) as function of active muscle length (l_ma_). Proximal muscle fibers from the aponeurotomized and recovered GM show similar lengths as function of muscle length compared to the control muscle fibers (*p* = 0.73), indicating that the intervention had no effect on the proximal muscle fibers. **(B)** The length of distal muscle fibers (l_fa_ distal) as function of active muscle length. Distal muscle fibers from the aponeurotomized and recovered GM show similar lengths as function of muscle length compared to the control muscle fibers (*p* = 0.07), indicating that the intervention had no effect on the distal muscle fibers. Two-way mixed ANOVA (with repeated measurements for muscle length) was used to investigate if l_fa_ of the aponeurotomized group was different to that of the control and sham operated. All values are presented as mean and either plus or minus SEM; control *n* = 10, sham *n* = 6, AT & recovery *n* = 6.

ANOVA showed that the proximal fiber length increased with increasing muscle length in all three treatments (*p* < 0.001; Figure 3A). Neither a significant main effect of treatment nor an interaction effect between fiber length and treatment was found (Figure 3A). This indicates that, after recovery from aponeurotomy, the length of muscle fibers located proximally from the created gap increased as a function of increasing muscle length undistinguishable from control GM.

#### Effects of Aponeurotomy and Recovery on Distal Muscle Fibers

ANOVA showed that the distal fiber length increased with increasing muscle length in all three treatments (*p* < 0.001). Neither a significant main effect of treatment nor an interaction effect between fiber length and treatment was found (Figure 3B). However, note that the curve describing the recovery from aponeurotomy group is located well below those of the other groups; however, this could not be shown to be significant.

### Effects on Serial Number of Sarcomeres After Recovery From Aponeurotomy

#### Effects on Serial Number of Sarcomeres Within Muscle Fibers Due to Different Treatments

**Figure 4:**
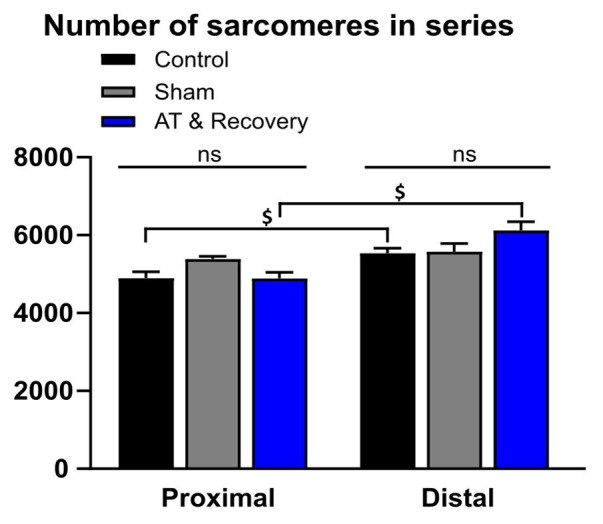
Effects of aponeurotomy plus recovery from aponeurotomy on the number of serial sarcomeres within muscle fibers. (1) Treatment differences. In the aponeurotomized plus recovered GM, the number of sarcomeres in series was similar to that of the control group (straight lines above the bar graphs indicate that both proximally and distally located fibers, the serial sarcomere numbers were similar between treatments) (*p* = 0.179). This may indicate either a lack of adaptation number of serial sarcomeres within distal muscle fibers or two opposing effects approximately compensating for each other (i.e., local protein degradation at one end and synthesis at the other end of the same muscle fiber). (2) Differences in the number of sarcomeres in series within muscles (proximal vs. distal fibers differences). Within muscle differences are indicated with “$.” Distal muscle fibers of the aponeurotomy plus recovery group and control group had more serial sarcomeres compared to proximal fibers (*p* = 0.001 and *p* = 0.017, respectively). It is unknown why in the sham proximal and distal fibers, the number of sarcomeres in series is similar, as from literature it is known that in GM distal fibers have higher numbers of sarcomeres in series. A two-way mixed ANOVA was used to check for differences in serial sarcomere number between treatments and within muscle location (proximally vs. distally located fibers). All values are presented as mean and plus SEM; control *n* = 10, sham *n* = 6, AT & recovery *n* = 6.

ANOVA showed no significant main effect of treatment on the number of serial sarcomeres, which indicates that, after recovery, the (absolute) serial numbers of sarcomeres in the proximal and distal fibers were not changed with respect to the control group (Figure 4).

#### Serial Number of Sarcomeres Within Fibers at Different Locations Within Muscle

ANOVA showed a significant effect of muscle location (proximal vs. distal) on the number of serial sarcomeres. Within distal muscle fibers, the number of serial sarcomeres was higher than within proximal fibers (*p* < 0.001). Moreover, an interaction effect between the fiber location (proximal vs. distal) and treatment was present for a series of sarcomere number (*p* < 0.05). *Post hoc* analysis showed in recovery and control groups that distal fibers contained 20 and 8% more sarcomeres in series, respectively, than proximal fibers (*p* < 0.001 and *p* < 0.02, respectively; Figure 4). In the sham-operated group, distal muscle fibers had similar numbers of sarcomeres in series as proximal fibers.

### Effects After Recovery From Aponeurotomy on Fiber Mean Sarcomere Length at Different Locations Within the Muscle

#### Effects Within Proximal Muscle Fibers

**Figure 5:**
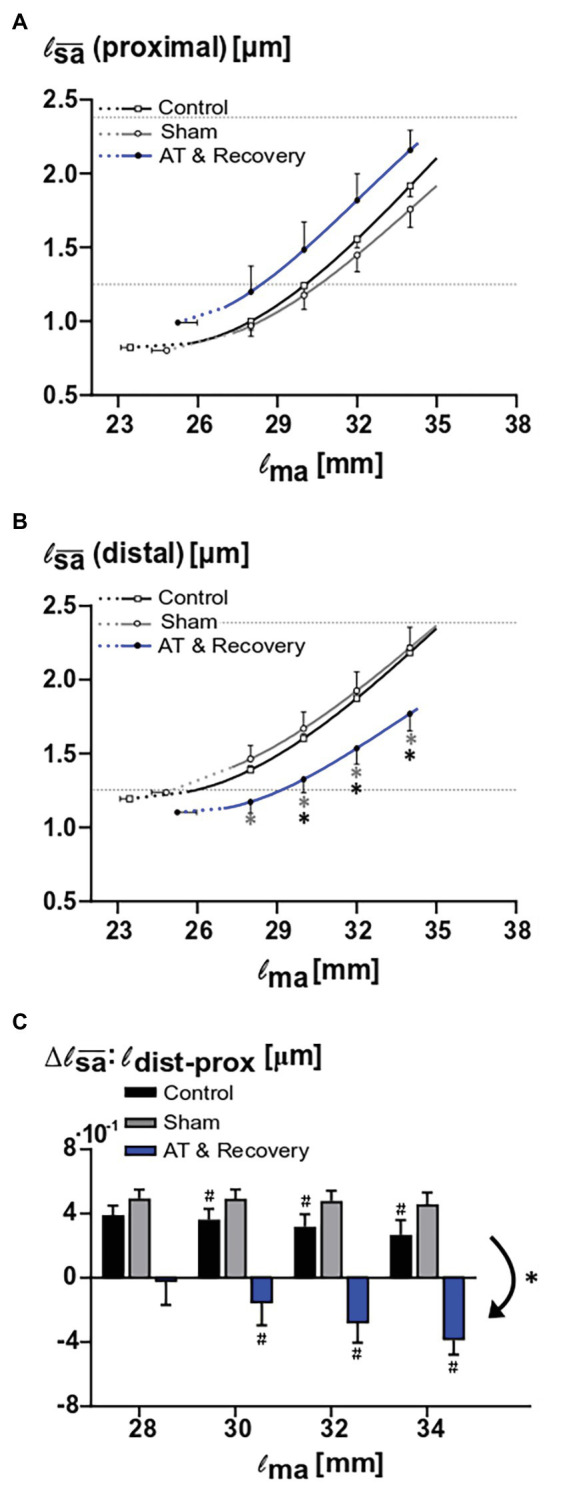
Effects of aponeurotomy plus recovery on fiber mean sarcomere lengths. **(A)** Proximal muscle fiber mean sarcomere length (l_$\bar{sa}$_ proximal) plotted as function of active muscle length. The aponeurotomy plus recovery had no significant effect on the active sarcomere lengths (*p* < 0.126). This indicates that the contribution of the proximal muscle fibers to muscle active force was not affected by the intervention. **(B)** Distal muscle fiber mean sarcomere length (|_$\bar{sa}$_ distal) as function of active muscle length. Differences between the aponeurotomy recovery and control group and sham group are noted with “*” (*p* = 0.026 and *p* = 0.018, respectively). Results show that because of the intervention, in distal fibers, the functioning fiber mean sarcomere length was decreased. This indicates that the contribution of these distal fibers to muscle force was also decreased. All values are presented as mean and either plus or minus SEM. Fiber mean sarcomere lengths as function of muscle length are plotted as continuous lines, whereas dashed lines indicate extrapolated parts of that curve to the estimated muscle active slack length. For reference, dotted horizontal lines indicate slack and optimum sarcomere length for untreated GM (Zuurbier et al., 1995). **(C)** Comparison of proximodistal differences in fiber mean sarcomere length between aponeurotomy plus recovery group and control group. Delta proximodistal fiber mean sarcomeres lengths of the aponeurotomy plus recovery group, sham, and control group are compared at different muscle lengths. The aponeurotomy plus recovery group shows a reversed distribution (i.e., negative distribution with respect to the control and sham group) (*p* = 0.001 for both *post hoc* comparisons), which increases with increasing muscle length (i.e., becomes more negative) within 28–30 mm (*p* = 0.015). In the aponeurotomy plus recovery group, the distribution is reversed compared to the control and sham group; however, total distribution is similar. Reversed distribution is noted with “*” and within effects (i.e., different distribution as function of muscle length) in the aponeurotomy plus recovery are noted with “#.” Two-way mixed ANOVA (with repeated measurements for muscle length) was used to check if the |_$\bar{sa}$_ (of proximal and distal muscle fibers, A and B) were different between groups. Control *n* = 10, sham *n* = 6, AT & recovery *n* = 6.

ANOVA showed that the proximal fiber mean sarcomere length increased with increasing muscle length in all three groups (*p* < 0.001). After recovery from aponeurotomy, for proximal muscle fibers, the curve relating muscle length and fiber mean sarcomere lengths was located well above those of sham and control group (Figure 5A). However, ANOVA did yield neither significant main effects nor an interaction.

#### Effects Within Distal Muscle Fibers

For distal muscle fibers, ANOVA showed a significant main effect of muscle length on fiber mean sarcomere lengths (*p* < 0.001), as well as a significant interaction between muscle length and treatment (*p* < 0.05).

*Post hoc* analysis showed that over a muscle length range of 28–34 mm, the fiber mean sarcomere lengths after recovery from aponeurotomy were about 20% smaller than that of the sham-operated group (*p* < 0.05). *Post hoc* analysis also showed that over a muscle length range of 30–34 mm, the fiber mean sarcomere lengths after recovery from aponeurotomy were 16–20% smaller than that of the control group (*p* < 0.05; Figure 5B).

It should be noted that even after 6 weeks of recovery from aponeurotomy, distal fibers still operate at quite low fiber mean sarcomere lengths. If this should have been the sole effect after recovery, one would expect effects on the length-force characteristics due to enhanced fiber mean sarcomere length distribution.

### Effects After Recovery From Aponeurotomy on Changes in Distribution of Fiber Mean Sarcomere Length

ANOVA showed a main effect of treatment on proximodistal differences of fiber mean sarcomere lengths (*p* < 0.001). After recovery from aponeurotomy, the sign of this proximodistal difference was reversed compared to the control as well as to the sham-operated groups (*p* < 0.001; Figure 5C). However, the absolute value of the range of fiber mean sarcomere length as a function of muscle length was similar between the groups, despite the fact that the locations of high and low lengths are reversed.

ANOVA showed a main effect of muscle length on proximodistal differences of fiber mean sarcomere lengths (*p* < 0.001), as well as an interaction effect between treatment and muscle length (*p* < 0.005). *Post hoc* analysis located the differences in proximodistal fiber mean sarcomere length differences between treatments: The control groups showed a significant decrease in distribution as muscle length was higher (*p* < 0.05). Therefore, these proximodistal differences should be considered as a primary (i.e., always present distribution). For the sham-operated group, the range proximodistal differences in fiber mean sarcomere length remained similar at higher muscle length. However, it should be noted that, after recovery from aponeurotomy, that situation was quite different: at low muscle lengths, the proximodistal difference of fiber mean sarcomere length was low or absent. However, at high muscle lengths, enhanced proximodistal differences of this variable developed (i.e., enhanced distributions of fiber mean sarcomere lengths; *p* < 0.05). This distribution, secondary to increased muscle length, attained levels similar in absolute value, but opposite in sign compared to those of the control sham-operated group (Figure 5C).

**Figure 6:**
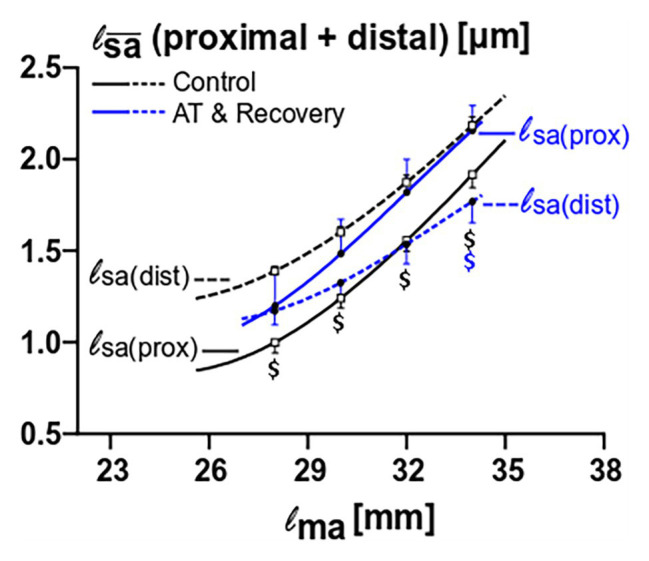
Fiber mean sarcomere length comparison between proximal and distal muscle fibers for the control versus the aponeurotomy plus recovery group. Note that in control muscle, proximal fibers have lower mean sarcomere length than proximal fibers; these differences are indicated with black “$” (*p* = 0.015), whereas in aponeurotomy plus recovery group the lines are reversed (proximal fibers have higher mean sarcomere lengths than distal fibers), and differences are indicated with blue “$” (*p* = 0.015). All values are presented as mean and either plus or minus SEM; Two-way mixed ANOVA (with repeated measurements for muscle length) was used to check if the mean fiber sarcomere lengths were different between groups. Control *n* = 10, AT & recovery *n* = 6.

ANOVA showed that there was significant interaction on fiber mean sarcomere length between factors muscle fiber location and treatment. For proximal fibers, the fiber mean sarcomere length after recovery was not found to be significantly different than that of the control group. In contrast, for distal fibers after recovery, the fiber mean sarcomere length was lower than that of the control group (*p* < 0.01; Figure 6).

ANOVA also showed significant interactions of muscle fiber location, muscle length, and treatment on fiber mean sarcomere length. After recovery from aponeurotomy, values for fiber mean sarcomere length of proximally located fibers were higher than in distally located fibers (*p* < 0.01), however, significance could only be shown at a high muscle length (i.e., at 34 mm). In contrast, in the control group, distally located fiber mean sarcomere length was higher than that of proximally located fibers at all lengths (*p* < 0.05; Figure 6).

These results indicate that the primary distribution of fiber mean sarcomere length, as found in the control group, is present in similar quantity at all muscle lengths, whereas after recovery the secondary distribution of fiber mean sarcomere length increases with increasing muscle length, yielding a proximodistal difference of this variable of opposite sign.

### Muscle Length-Force Characteristics

**Figure 7:**
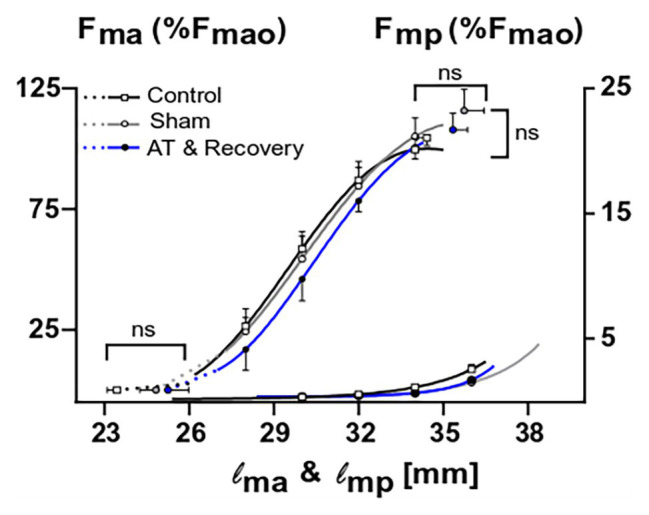
The combined effects of aponeurotomy plus recovery from aponeurotomy on the muscle active and passive length-force characteristics. Normalized active and passive muscle forces (*F*_ma_ and *F*_mp_, respectively) as a function of muscle length (l_m_) are plotted in continuous lines. Extrapolated forces, ranging from the lowest measured muscle lengths to the estimated muscle active slack length, are plotted as dashed lines. After aponeurotomy plus recovery, active muscle force and length-force characteristics were similar to those of control GM. The passive length-force curve of the aponeurotomy plus recovery group was also similar to that of the control group. This indicates that after 6 weeks of recovery, the intervention had no effect on the muscle length range of active or passive force exertion, neither had the aponeurotomy plus recovery an effect on the force generating capacity. Two-way mixed ANOVA (with repeated measurements for muscle length) was used to check if (normalized) muscle force was similar between groups. Additionally, a one-way ANOVA was used to check for differences between the optimum muscle force, optimum muscle length, and slack length. All values are presented as mean and either plus or minus SEM; control *n* = 10, sham *n* = 6, AT & recovery *n* = 6.

Figure 7 shows muscle active and passive length-force characteristics for controls, sham-operated ones, and after recovery from aponeurotomy. Length-active force curves were similar for these groups. Active slack and optimum muscle lengths, as well as optimal force, could not be shown to differ significantly between treatments.

Also, muscle length-passive force characteristics were similar for the control, sham, and recovery from aponeurotomy groups (Figure 7). These results indicate that neither the active nor the passive length-force relationship was changed after recovery from aponeurotomy, despite altered fiber mean sarcomere lengths distributions.

## Discussion

**Figure 8:**
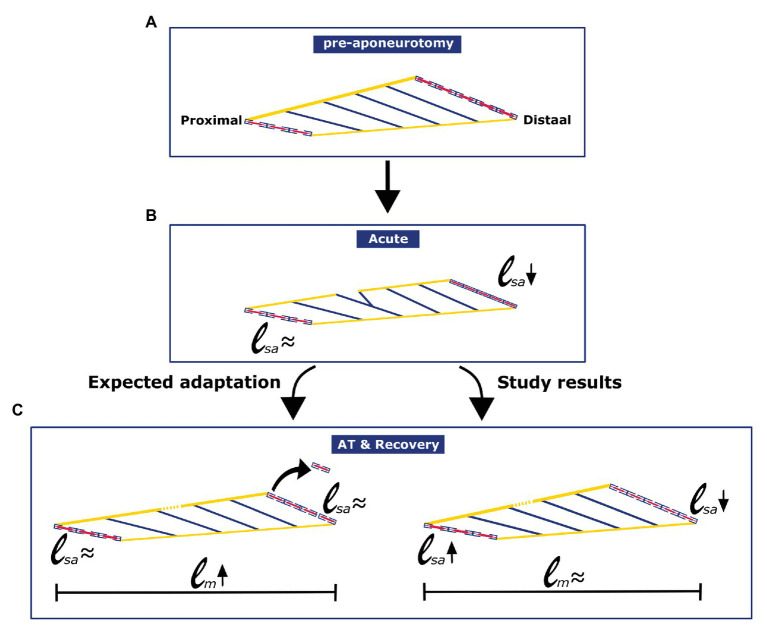
Schematic figure illustrating the effects of aponeurotomy acutely and in long term, as was expected, and as was found in current study. **(A)** Pre-aponeurotomy: Distally located muscle fibers have higher number of serial sarcomeres than proximally located fibers. **(B)** Acute: Acutely after the aponeurotomy, distal muscle fibers mean sarcomere length decreased, while the length of proximal fibers was unchanged (Jaspers et al., 1999). **(C)** AT & Recovery: Adaptation after aponeurotomy and recovery was not as expected from the general rules of adaptation. In distally located muscle fibers, numbers of sarcomeres in series were similar to that of fibers from the control group. However, distal muscle fibers mean sarcomere length remained low.

The present results, describing effects of a period of recovery (6 weeks) from proximal aponeurotomy, show that GM muscles have *in situ* length-force characteristics similar to controls. Furthermore, fibers located distally from the aponeurosis gap showed reduced fiber mean sarcomere lengths. For fibers located proximally from the aponeurosis gap, no significant effect on mean sarcomere length could be shown. For fibers located proximally, as well as distally from the aponeurotomy gap, the number of sarcomeres in series was not different from that of the control GM (see Figures 8A-C for schematic summary). Within, the control group, distal fibers had higher mean sarcomere lengths. In contrast, after recovery from aponeurotomy, proximal fibers contained the highest mean sarcomere lengths, indicating a reversal of the proximodistal difference of this distribution.

### Aponeurotomy Acutely Alters Muscle Geometry and Related Functional Characteristics

For adaptation aspects of recovery from aponeurotomy, we need to consider the acute effects imposed on the muscle due to the aponeurotomy, as they form the initial condition from which the initial adaptations in recovery occur.

Effects of acute intramuscular tearing due to proximal aponeurotomy were described by Jaspers et al. (1999). Muscle fibers attaching distally from the created aponeurosis gap lose their myotendinous connections to the muscle’s origin. Fibers of the acutely aponeurotomized GM located proximally from that gap had similar mean sarcomere lengths as those from intact GM for increasing muscle length. However, acutely after aponeurotomy, lengths of fibers located distally from the aponeurosis gap showed only minimally increasing mean sarcomere lengths with increasing muscle lengths. The unchanged proximal fibers mean sarcomere length combined with the minimal increase of the distally located fibers resulted in temporarily reduced muscle force and small increments of slack and optimum lengths (Jaspers et al., 1999).

In finite element modeling (FEM) of acute effects of aponeurotomy, serial sarcomeres from fibers that lost their myotendinous connection with the origin of the muscle were shown to attain extreme low length. This latter was mostly the case in the most distal part of the most distal muscle fibers (i.e., part of the fiber, which is close to the Achilles tendon; Yucesoy et al., 2007, 2013). The FEM results also indicate that the local fiber direction strain, within fibers located distally from the aponeurotomy gap, was lower than that in proximal fibers. Most sarcomeres within the modeled distal fiber population shortened to their active slack length. However, even the most distal sarcomeres within the distal muscle fibers could still contribute to muscle force. This was possible as their force was transmitted *via* myofascial connections to fibers with intact myotendinous connections at both ends (within the proximal population). This model indicates that serial sarcomeres length distribution (within muscle fibers) was increased by the intervention, which should lead to an increase in length range of active force exertion. Note that the FEM studies referred to show some noticeable differences with our present data (i.e., acute effects are modeled vs. our recovery effects, as well as extra muscular myofascial connections present in the model vs. their absence in our situ muscles). Therefore, some different results (e.g., a shift of active muscle force to higher muscle length in combination with lower force) are likely to be explained. However, the model provides a valuable help for understanding how parallel and serial distribution of sarcomere length affects the muscle and its morphological and physiological characteristics acutely after aponeurotomy.

### Effects of Recovery

During subsequent recovery, new collagenous (scar) tissue fills the gap in the aponeurosis and within the muscle (Jaspers et al., 1999, 2002). As a consequence of such effects on the aponeurosis, fibers located distally from the previous gap in the aponeurosis operate at lower lengths due to (1) a longer aponeurosis, and (2) the new collagenous material filling up the gap of the aponeurosis is more compliant than that of the unaffected part of the aponeurosis (Jaspers et al., 2005).

In contrast to the acute effects of aponeurotomy (Jaspers et al., 1999, 2002), our present results show that after a period of recovery from aponeurotomy, muscle length-force characteristics were similar to those of control muscles. It is obvious that this result may need clinical attention. In fibers distally from the aponeurotomy gap, because of enhanced compliance of the aponeurosis, the distal fiber mean sarcomere length decreased.

In principle, the presence of the parallel distribution of fiber mean sarcomere lengths increases the muscle length range of active force exertion; however, this happens at the expense of a lower optimum force (e.g., Willems and Huijing, 1994).

However, effects of such changes were not present in GM muscle length-force characteristics after recovery from aponeurotomy (present results). The explanation is likely to be a reversed proximodistal difference of mean sarcomere lengths. Our present results show that at low muscle lengths, distal muscle fiber mean sarcomere length distribution was absent or low. However, a secondary distribution of mean fiber sarcomere length develops at higher muscle lengths. Apparently the changes in mean sarcomere lengths are of similar size but opposite in nature. Because of this, changes of active slack and optimum length were very small or absent, causing the muscle length range of active force exertion to be similar to that of the intact GM.

### Adaptation of the Muscle to the New Conditions After Aponeurotomy

The rules of adaptation of the serial number of sarcomeres according to Herring et al. (1984) seem to be widely accepted. This rule yields the view that adaptation is such that muscle optimum length is adapted for it to occur at the length at which the muscle operates most frequently or joint angle at which the muscle is most frequently active (or experimentally has been immobilized). For muscles with a low degree of pennation, it has been accepted generally (Tabary et al., 1972; Williams and Goldspink, 1973; Herring et al., 1984; Heslinga et al., 1995; Koh and Herzog, 1998) that adaptation of the serial number of sarcomeres causes that effect. Because most sarcomeres of the distal fiber population are kept at low lengths after aponeurotomy and even after recovery (present results), one would expect the number of sarcomeres in series of such distal fibers to decrease. However, our present results show this not to be the case.

In contrast, in highly pennate muscles, changes of optimum muscle length are achieved also by the effects of hypertrophy or atrophy (i.e., changes in the physiological cross-sectional area; Swatland, 1980; Heslinga et al., 1992 1995; Heslinga and Huijing, 1993; Weide et al., 2015). However, after recovery from aponeurotomy there are no indications that this effect of altered physiological cross-sectional area plays a role, as optimal force cannot be distinguished between the groups.

Two plausible explanations for the lack of changes in serial sarcomere number within muscle fibers after recovery from aponeurotomy are described below:

(1) In *ex vivo* culture of single muscle fibers, overall (positive or negative) strains did not induce changes in the serial of sarcomere number of *Xenopus* (Jaspers et al., 2004). The discrepancy between these *ex vivo* results and *in vivo* as well as *in situ* results may be explained by the serial sarcomere length heterogeneity within muscle fibers (Pappas et al., 2002; Ahn et al., 2003). Interaction of muscle fibers with neighboring fibers are most likely to be responsible for the unequal force exertion on the proximal and distal tendon (Huijing and Baan, 2001; Maas et al., 2001; for a review of results see Huijing, 2009) and heterogeneity of sarcomere strain within the single muscle fiber (Yucesoy et al., 2003). In *ex vivo* conditions, the target single muscle fiber can only interact with (the remainder of) its endomysium. Therefore, applied strain on single muscle fibers is more likely to cause global values for end-to-end fiber strains.
In contrast, by applying strain on a whole muscle, local fiber strains at one end of a muscle fiber may be low, whereas at the other end, or even at other parts of the same fiber, it may be high. This may create signals causing local protein degradation at one end and synthesis at the other end of the same muscle fiber, with possibly no net change of serial sarcomere number of that whole muscle fiber. The presence of shear forces (i.e., parallel forces but of opposite direction applied onto muscle fibers) is likely to play an important role: such forces applied onto the most distal parts of fibers located distally from the aponeurotomy gap prevent these fibers from fully shortening to their active slack length. Such features are also apparent from the fact that such distal parts of distally located fibers are subject to lengthening with increasing muscle length. Moreover, fluid shear stress applied to myotubes *in vitro* has been shown to enhance the production of nitric oxide (NO; Juffer et al., 2014). NO is involved in the activation of signaling pathways for muscle hypertrophy (Ito et al., 2013), as well as for adaptation of the number of sarcomere in series (Koh and Herzog, 1998). These results lead to the hypothesis that after aponeurotomy enhanced shear loading of the extracellular endomysium in the distal part could have induced a signal for protein synthesis, which may have counterbalanced signals for protein degradation.
(2) *In vivo*, because of intact extramuscular connections with surrounding structures, fibers located distally from the aponeurotomy gap are prevented from shortening as much as occurs *in situ*. This indicates that muscle force, muscle geometry, and other functional characteristics are dependent on extramuscular or intermuscular myofascial connections and that dissection of surrounding structures will remove the changes caused by interaction with a higher level of organization.

The lack of adaptation after aponeurotomy and recovery, as seen in this study, may be explained by either effect or a combination of effects described above.

### Limitations of This Study

This study has some obvious limitations that should be taken into account interpreting the results. Rats used in this current study did not suffer from spasticity. Therefore, the current results reflect adaptive processes in unaffected muscle, whereas children with spastic paresis are known to develop differently from typically developing children (e.g., review of Gough and Shortland, 2012). Consequently, extrapolation of our present results to this human condition should be handled with care.

After *in situ* measurements, muscles and some of its fibers were isolated in order to allow counting of sarcomeres in series. However, we lack estimates of muscle volume and physiological cross-sectional area. Tibia length was not measured either, so that effects of bone growth could not be taken into account, and normalization of lengths for this variable could not be performed. As a consequence, we only present the length-force curves normalized for optimum muscle force of GM muscles of the control group.

### Clinical Applications

Knee and ankle flexors, considered overly short, are often treated using aponeurotomy during multilevel surgery in children or adolescents with cerebral palsy. Moreover, this procedure is also used in adults with such problems, clinically referred to as muscle contracture (e.g., in plantar fasciitis, Achilles tendinopathy, metatarsalgia, and forefoot ulceration). The muscle-tendon complex is lengthened in several different procedures by extramuscular aponeurotic lengthening (Vulpius and Stoffel, 1924; Strayer, 1950 1958; Baker, 1954) or intramuscular variant of it (Baumann and Koch, 1989). The surgical principle of aponeurotomy has also been applied to other muscles and used in modified techniques (intramuscular lengthening, fibrotomia). As effects of extramuscular and intramuscular aponeurotomy are quite different, the results of the present study should only be used as an idea-generating feature for the latter.

Results of animal studies (Brunner et al., 2000; Jaspers et al., 2005) regarding intramuscular aponeurotomy have shown that the created gap in the aponeurosis is filled with new compliant connective tissue, and the gap in the muscle is filled by new muscle fibers. *In vivo*, the effects of such adaptation on muscle geometry and functional characteristics are unknown. Moreover, apart from possible performance of multiple aponeurotomies, *in vivo* the intervention and dissection of surrounding connective tissue is minimized compared to rodent studies. The possible effects on muscle geometry and related functional characteristics of these differences are also unknown.

Acute effects of aponeurotomy (Jaspers et al., 1999) and present results after longer-term periods of recovery show that for distally located muscle fibers, the fiber mean sarcomere length is and remains reduced. It is reasonable to hypothesize that these distal fibers and their spindles attain similar levels of stretch than before the aponeurotomy, but at a higher muscle length (e.g., Hagbarth et al., 1975; Wilson et al., 1997). It has been hypothesized that reducing stretch on muscle spindles at low muscle length may reduce the velocity-dependent overactive stretch reflex and allow the spastic muscle to attain a greater muscle length range (Gracies, 2005). On the other hand, effects of increased sarcomere length in proximal muscle fibers may diminish or negate such effects. It is conceivable that these features may play a role in the relatively high incidence of recurrent contracture after surgical interventions.

Taking into account the limitations of the present study described above, one feature requires most clinical attention: in contrast to the acute effects of aponeurotomy (Jaspers et al., 1999), our present results show that after a period of recovery from aponeurotomy, the GM has similar muscle length-force characteristics to those of control muscles. This indicates that the surgical aim of the intervention, namely, increasing the length range of active force exertion of the target muscle, may not be accomplished at the longer term.

## Conclusion

This study shows that after recovery from aponeurotomy, fibers that were kept at low fiber mean sarcomere lengths did not adapt their serial sarcomere number. A reversed proximodistal difference of fiber mean sarcomere length was found after recovery due to altered mechanical conditions originating from altered aponeurosis properties. It should be noted that the altered mechanical conditions affect not only the distal fiber population, but also the proximal fiber population within GM. It is hypothesized that because of the effects of this reversal, the net effect of the intervention on the muscle length range of active force exertion is zero. It seems that the rules of adaptation as far as we know them are not applicable to the conditions imposed in this study and may be in need of review. Further research is necessary in order to understand how the intervention can be optimized, with the purpose of reducing the number of recurrent cases in children with spasticity.

## Data Availability Statement

The datasets generated for this study are available on request to the corresponding author.

## Ethics Statement

The animal study was reviewed and approved by Veterinary office of Basel-Stadt in Switzerland. Experiments were performed in accordance with the Swiss law on animal use and welfare and was approved by the Veterinary office of Basel-Stadt.

## Author Contributions

RJ, JP, RB, and PH conceived and designed research. RJ, JP, RB, and GB performed experiments. CR and RJ analyzed data. CR, PH, and RJ interpreted results and drafted manuscript. CR and GB prepared figures. RB, JP, and GB revised manuscript. All authors contributed to the article and approved the submitted version.

### Conflict of Interest

The authors declare that the research was conducted in the absence of any commercial or financial relationships that could be construed as a potential conflict of interest.
